# Pulmonary sequelae of SARS-CoV-2 infection: a narrative review of long-term functional, radiological, and clinical outcomes in COVID-19 survivors, with a regional focus on Saudi Arabia

**DOI:** 10.3389/fphys.2026.1916914

**Published:** 2026-09-02

**Authors:** Fatimah Ali Zarbatan, Khaled Alawam, Ali Hakamy

**Affiliations:** 1 Respiratory Therapy Program, Inaya Medical Colleges, Riyadh, Saudi Arabia; 2 Respiratory Therapy Department, King Fahd Central Hospital, Jazan Medical Cluster, Jazan, Saudi Arabia; 3 Nursing Department, College of Nursing and Health Sciences, Jazan University, Jazan, Saudi Arabia

**Keywords:** COVID-19, post-acute sequelae, pulmonary fibrosis, pulmonary function tests, respiratory rehabilitation

## Abstract

The severe acute respiratory syndrome coronavirus 2 (SARS-CoV-2) pandemic has left an enduring legacy of pulmonary morbidity among millions of survivors worldwide. While the acute phase of COVID-19 has been extensively characterized, the long-term pulmonary sequelae collectively termed post-acute sequelae of SARS-CoV-2 infection (PASC) or long COVID remain incompletely understood, particularly across diverse geographic and demographic populations. This narrative review synthesizes current evidence on the long-term pulmonary consequences of SARS-CoV-2 infection, with emphasis on functional, radiological, and clinical outcomes in survivors. Literature was identified through systematic searches of PubMed, Scopus, and Google Scholar covering publications from January 2020 to September 2025, using pre-specified search terms related to long COVID pulmonary outcome; studies involving adult survivors with objectively assessed pulmonary outcomes were prioritized. This review addresses the pathophysiological mechanisms underlying pulmonary injury, the definition and clinical spectrum of long COVID, the trajectory of pulmonary function test (PFT) abnormalities—particularly reductions in diffusing capacity for carbon monoxide (DLCO) and radiological findings detected on high-resolution computed tomography (HRCT). Risk factors for persistent pulmonary dysfunction, the impact on health-related quality of life (HRQoL) and functional capacity, emerging evidence supporting structured pulmonary rehabilitation, and the specific context of Saudi Arabia and the Jizan region are also examined. A particular focus on the Arabian Peninsula is warranted given that regional populations carry a high burden of metabolic comorbidities including obesity, diabetes mellitus, and hypertension that may amplify pulmonary vulnerability to SARS-CoV-2 yet remain substantially underrepresented in the current literature. The available evidence suggests that impaired gas exchange most consistently reflected by reduced DLCO may represent the most prevalent long-term pulmonary consequence of COVID-19, with restrictive ventilatory patterns predominating over obstructive defects. Significant gaps persist regarding the long-term trajectory of these abnormalities, population-specific risk stratification, and the efficacy of targeted rehabilitation. Structured, longitudinal, multicenter research incorporating standardized PFT protocols is urgently needed to address these unresolved questions and guide evidence-based post-COVID pulmonary care.

## 1 Introduction

The emergence of coronavirus disease 2019 (COVID-19), caused by severe acute respiratory syndrome coronavirus 2 (SARS-CoV-2), represented an unprecedented challenge to global public health. First identified in Wuhan, China, in December 2019, the virus rapidly disseminated across all continents, prompting the World Health Organization (WHO) to declare a Public Health Emergency of International Concern in January 2020 and a global pandemic on March 11, 2020 (World Health Organization, 2025a). By September 2025, SARS-CoV-2 had infected over 778 million individuals and caused more than 7.1 million deaths worldwide (World Health Organization, 2025b), establishing it as one of the most consequential infectious diseases in the modern era.

In the early phases of the pandemic, scientific and clinical attention was appropriately directed toward characterizing the acute disease syndrome, developing diagnostic tools, and deploying therapeutic and preventive interventions to reduce acute mortality. However, as the global population of recovered individuals grew to unprecedented numbers, a parallel and increasingly pressing concern emerged: the persistence of symptoms and organ dysfunction well beyond the resolution of acute illness.

This phenomenon, widely recognized as long COVID or post-acute sequelae of SARS-CoV-2 infection (PASC), is now understood to affect a substantial proportion of survivors. The Centers for Disease Control and Prevention (CDC) defines long COVID as a chronic condition persisting beyond three months following SARS-CoV-2 infection (Centers for Disease Control and Prevention (CDC), 2025). Among the organ systems implicated in post-acute sequelae, the pulmonary system occupies a position of particular clinical importance, given the virus’s primary tropism for the respiratory epithelium and the centrality of respiratory failure in determining acute disease outcomes.

Despite an expanding body of literature, critical questions regarding the long-term trajectory of pulmonary function impairment, the identification of at-risk populations, and the optimal management of post-COVID-19 pulmonary sequelae remain incompletely answered. This gap is especially pronounced in the Arabian Peninsula. In populations across Saudi Arabia and neighboring regions, the high prevalence of obesity, diabetes mellitus, and hypertension creates a distinctive risk profile that may substantially amplify susceptibility to severe COVID-19 and impair long-term pulmonary recovery yet these populations are largely absent from the high-quality longitudinal literature that currently informs clinical guidelines (Althumiri et al., 2021). Addressing this geographic and demographic gap is therefore both scientifically necessary and clinically urgent.

This narrative review was conducted through electronic searches of PubMed/MEDLINE, Scopus, and Google Scholar, covering the period from January 2020 to September 2025. Search terms were constructed around the following concepts: “SARS-CoV-2” OR “COVID-19” AND “long COVID” OR “post-acute sequelae” OR “PASC” AND “pulmonary function” OR “DLCO” OR “spirometry” OR “HRCT” OR “lung fibrosis” OR “respiratory rehabilitation” OR “Saudi Arabia”. The search was supplemented by manual review of reference lists from identified systematic reviews and meta-analyses. Priority was given to peer-reviewed original research articles, systematic reviews, and meta-analyses involving adult survivors (≥18 years) of SARS-CoV-2 infection with objectively assessed pulmonary outcomes including spirometry, body plethysmography, DLCO, or HRCT. Case reports, animal studies, and pediatric-only populations were excluded. Given the narrative scope of this review, a formal PRISMA-based systematic search was not conducted; however, source selection was guided by relevance, quality, and recency of available evidence.

## 2 Epidemiology and global burden of SARS-CoV-2

COVID-19 was first announced in December 2019 in Wuhan, China, and within months had spread to every continent (World Health Organization, 2025a). As of September 2025, the WHO documented over 778 million confirmed infections and more than 7.1 million deaths globally (World Health Organization, 2025b). These figures, however, capture only the acute-phase mortality and substantially underrepresent the true disease burden borne by hundreds of millions of survivors who continue to experience long-term health consequences, particularly in the respiratory system.

The socioeconomic ramifications of SARS-CoV-2 have been profound. Research has demonstrated that many COVID-19 survivors face reduced physical function and persistent multisystem complications including respiratory, cardiovascular, and neurocognitive disorders that necessitate sustained healthcare engagement (Althumiri et al., 2021). Disability-adjusted life years (DALYs) attributable to COVID-19, particularly in the context of long-term sequelae, underscore the magnitude of this burden beyond mortality statistics alone (Nurchis et al., 2020; Donnelly et al., 2021).

Studies examining post-discharge health outcomes have consistently demonstrated that the disease burden extends well beyond the acute phase: a systematic review and meta-analysis of 91 studies and 283,468 patients reported a hospital readmission rate of 8.97% within three months post-infection, rising to 10.34% over one year, with substantially elevated rates in elderly patients (15.28%) and those with comorbidities (19.63%) (Ramzi, 2022). A systematic review with follow-up periods of up to three years further found that approximately 20% of survivors reported at least one persistent symptom, with dyspnea, fatigue, and insomnia predominating (Rahmati et al., 2025). In Saudi Arabia, the impact has been particularly notable; data from 2021 identified SARS-CoV-2 as the second leading cause of death in female patients (29.3 deaths per 100,000) and the third in male patients (25.3 per 100,000) (World Health Organization, 2025c).

## 3 Pathophysiology of pulmonary injury: viral mechanisms and the inflammatory cascade

Understanding the long-term pulmonary sequelae of SARS-CoV-2 requires an appreciation of the mechanisms through which the virus induces and sustains pulmonary injury. SARS-CoV-2 exhibits a high binding affinity for angiotensin-converting enzyme 2 (ACE2) receptors and transmembrane serine protease 2 (TMPRSS2), both of which are abundantly expressed on type II alveolar pneumocytes and airway epithelial cells, facilitating efficient viral entry (Aguiar et al., 2020; Hoffmann et al., 2020). In addition to these primary receptors, the virus may utilize alternative entry pathways involving CD147, GRP78, and Cathepsin L1, further activating downstream pro-inflammatory signaling cascades (Vaz de Paula et al., 2022).

Upon cellular entry and replication, SARS-CoV-2 triggers dysregulated release of pro-inflammatory cytokines, a pathological state termed the cytokine storm producing diffuse alveolar damage (DAD), characterized by alveolar epithelial injury, endothelial dysfunction, vascular leakage, and hyaline membrane formation, all of which impair alveolar-capillary gas exchange (Vaz de Paula et al., 2022).

A clinically critical consequence of SARS-CoV-2-mediated endothelial injury is the development of persistent pulmonary microthrombosis. The virus’s direct invasion of vascular endothelial cells, combined with the pro-coagulant state generated by cytokine-driven endothelial activation, promotes *in situ* thrombus formation within the pulmonary microvasculature. *In vivo* evidence of microvascular thrombosis in severe COVID-19 has been directly demonstrated through histopathological and immunological studies (do Espírito Santo et al., 2020; Nicolai et al., 2020). Immunothrombotic dysregulation characterized by platelet-neutrophil aggregates and neutrophil extracellular traps within pulmonary vessels is significantly associated with respiratory failure and coagulopathy (Vanderbilt et al., 2025). More recently, ischemic endothelial necroptosis has been identified as a mechanism of microvascular obstruction through red blood cell hemolysis in COVID-19 angiopathy (Whitcomb et al., 2026). Persistent pro-thrombotic platelet activation and elevated thrombotic analytes have further been documented in PASC, suggesting that the microvascular pro-coagulant state extends well beyond the acute phase (Kamal et al., 2025). This microvascular occlusion creates regions of ventilated but non-perfused lung parenchyma, producing ventilation/perfusion (V/Q) mismatch that selectively impairs gas diffusion across the alveolar-capillary membrane. This mechanism explains a central clinical observation in post-COVID-19 survivors: DLCO which measures the transfer efficiency of carbon monoxide across the alveolar-capillary unit is frequently and substantially reduced even in patients with relatively preserved spirometric values (FEV_1_, FVC). Because spirometry measures bulk airflow rather than membrane diffusion capacity, it is insensitive to the focal microvascular and interstitial damage that persists after the resolution of airway inflammation.

A critical molecular mediator linking acute inflammation to chronic fibrotic sequelae is transforming growth factor-beta (TGF-β). Immunohistochemical analysis has directly demonstrated TGF-β activation in COVID-19-associated pulmonary fibrosis, with TGF-β-driven fibroblast activation and myofibroblast transformation initiating collagen deposition and progressive fibrotic remodeling (Vaz de Paula et al., 2022).The development of acute respiratory distress syndrome (ARDS) which complicates approximately 23–33% of hospitalized COVID-19 cases (Tzotzos et al., 2020; Tsai et al., 2022) further amplifies structural lung injury through diffuse alveolar damage, organizing pneumonia, and fibroproliferative remodeling. COVID-19-associated pneumonia is an additional driver of long-term pulmonary morbidity: a 12-month follow-up study found HRCT abnormalities in 55% of patients who developed pneumonia, alongside persistent dyspnea (34.7%) and cough (29.5%) (Salem et al., 2021; Rosas et al., 2025).

In summary, the long-term pulmonary sequelae of COVID-19 arise from a convergence of pathological processes: microvascular microthrombosis generating V/Q mismatch; alveolar-capillary membrane disruption reducing diffusion capacity; TGF-β-mediated fibroproliferative remodeling restricting lung volumes; and incomplete alveolar epithelial regeneration sustaining structural impairment. Together, these mechanisms explain why DLCO reduction and restrictive ventilatory defects are the hallmark functional abnormalities observed in post-COVID-19 PFT assessments.

## 4 Defining long COVID: criteria, timeline, and the clinical spectrum

Despite extensive global research, the definition of long COVID have evolved considerably and continue to be refined. The CDC defines long COVID as a chronic condition developing after SARS-CoV-2 infection and enduring for more than three months, encompassing a wide range of symptoms across multiple organ systems (Centers for Disease Control and Prevention (CDC), 2025). Synonymous terminologies including PASC, post-COVID-19 syndrome, and post-acute COVID-19 reflect definitional heterogeneity rather than distinct clinical entities.

Studies have estimated that up to 80% of individuals infected with SARS-CoV-2 may develop one or more long-term symptoms (Lopez-Leon et al., 2021), and recent longitudinal data confirm these can persist beyond three years in a meaningful proportion of survivors (Rahmati et al., 2025). Critically, severity of acute disease is not a reliable predictor of long COVID; prospective studies demonstrates that persistent symptoms affect survivors across the spectrum of initial disease severity (Niebauer et al., 2023), including non-hospitalized individuals.

Among long COVID manifestations, persistent respiratory symptoms carry the greatest clinical significance from a pulmonary physiology standpoint. A cross-sectional systematic review of 25 observational studies including 5,440 patients identified chest pain in 89%, fatigue in 65%, and dyspnea in 61% of survivors (Cabrera Martimbianco et al., 2021; Dehlia and Guthridge, 2024). A prospective study following 150 patients for up to six months found that 75% had persistent symptoms, with exertional dyspnea and chronic fatigue in 62%, and that most survivors with dyspnea demonstrated objective reductions in pulmonary function (Niebauer et al., 2023).

Hospitalization and ICU admission substantially amplify this risk: a two-year longitudinal cohort of 650 hospitalized patients found that 73% had not fully recovered, with dyspnea and memory difficulty predominating in ICU survivors (Berentschot et al., 2024). A large-scale cohort of over 135,000 post-COVID individuals further confirmed that even non-hospitalized patients are not exempt from long-term pulmonary complications (Cai et al., 2024).

Pre-existing chronic lung disease (CLD) further potentiates the risk of post-COVID pulmonary sequelae, with asthma, COPD, and bronchiectasis conferring the highest risk in retrospective cohort data (Zhang et al., 2024). Conversely, SARS-CoV-2 infection itself may precipitate *de novo* CLD: a multicenter retrospective cohort of 69,632 participants reported a significantly higher incidence of new-onset chronic pulmonary conditions in COVID-19 survivors compared to uninfected controls, most notably pulmonary fibrosis (71%), pulmonary hypertension (16.25%), and emphysema (15%) (Henry et al., 2025). These bidirectional relationships underscore the need for vigilant respiratory surveillance.

Long COVID symptoms are embedded within a broader context of substantially impaired health-related quality of life (HRQoL). A systematic review and meta-analysis of 12 studies involving 4,828 participants found that 58% of post-COVID-19 patients experienced a decline in overall QoL, 36% reported mobility limitations, and 28% had difficulties with activities of daily living (Malik et al., 2022). EQ-5D-5L-based assessment (a standardized, five-dimension patient-reported outcome measure that rates mobility, self-care, usual activities, pain/discomfort, and anxiety/depression each on a five-level severity scale, generating a health utility score from 0 to 1) revealed that 41.5% experienced pain and discomfort, while 37.5% suffered from anxiety and depression. Fatigue and dyspnea were the most commonly reported and persistent symptoms, frequently accompanied by general weakness, myalgia, and sleep disturbances, particularly in previously hospitalized patients (Malik et al., 2022). These HRQoL deficits are not merely self-reported inconveniences; they reflect the functional consequence of underlying pulmonary impairment that limits cardiorespiratory reserve during daily activities.

## 5 Long-term pulmonary function impairment: evidence from PFT-based studies

Objective assessment of pulmonary function through standardized testing has been central to characterizing the long-term respiratory consequences of COVID-19. Among the various PFT parameters evaluated, the diffusing capacity of the lung for carbon monoxide (DLCO) has consistently emerged as the most sensitive and frequently impaired measure, reflecting ongoing alveolar-capillary membrane dysfunction even when spirometric values appear preserved.

A comprehensive systematic review and multicenter cohort confirmed that 40.2% of patients developed DLCO below 80% of predicted value within this same three-to-six-month window (Cornelissen et al., 2024). Studies performing PFTs within the first month post-infection have reported DLCO impairment in 27% to 52.6% of patients (Frija-Masson et al., 2020; Huang et al., 2020), while three-month assessments consistently identify impairment in 31% to 47.2% of survivors (Long et al., 2021; Sanchez-Ramirez et al., 2021; Torres-Castro et al., 2021). **Table 1** summarizes key PFT findings across follow-up milestones from published cohort and review studies.

**Table 1:** Summary of pulmonary function test (PFT) abnormalities in post-COVID-19 survivors across key follow-up time points.

Study/cohort	Follow-up	n	DLCO <80% (%)	Restrictive PFT (%)	Normal spirometry (%)
Frija-Masson et al. (2020) (Frija-Masson et al., 2020)	1 month	101 (independent cohort)	52.6	22.0	—
Huang et al. (2020) (Huang et al., 2020)	1 month	84 (independent cohort)	27.0	—	—
Cornelissen et al. (2024) (Cornelissen et al., 2024)	3–6 months	SR: 47 studies; Cohort n=229 (independent multicenter)	40.2	~39	~72
Thomas et al. (2021) [review] (Thomas et al., 2021)	3–6 months	Review: no original cohort	40–50	17–28	72–76
González et al. (2023) (González et al., 2023)	2 years	109 (independent; ICU survivors)	45.7	Restrictive dominant	—
Iversen et al. (2024) (Iversen et al., 2024)	2 years	58 (independent; mild COVID only)	22.4*	—	—
Han et al. (2024) (Han et al., 2024)	3 years	3-year follow-up of Wuhan discharge cohort†	49**	—	HRCT 36%
Ippoliti et al. (2025) (Ippoliti et al., 2025)	3 years	Independent HCW occupational cohort	—	—	FVC/FEV_1_ ↓ vs pre-COVID

DLCO, diffusing capacity of the lung for carbon monoxide, SR, systematic review, HCW, healthcare workers. *Mean % reduction from predicted. **Proportion achieving DLCO normalization. ^†^Han et al. (2024) is a longitudinal 3-year follow-up of the Wuhan COVID-19 discharge cohort originally reported by Huang C et al. (Lancet 2021) at 6 months, data are not independent from earlier timepoints of this cohort. Thomas et al. is a narrative review with no original cohort data.

↓, decreased.

These reductions do not appear to be uniformly transient. A two-year follow-up study demonstrated DLCO reduction of 22.4% at two years in mild COVID-19 patients, with FVC and FEV_1_ also continuing to decline (Iversen et al., 2024). At two years in a cohort of 109 hospitalized patients, 45.7% still exhibited reduced DLCO and 18.7% had moderate-to-severe impairment (González et al., 2023). A three-year longitudinal cohort demonstrated DLCO normalization in only 49% of survivors (Han et al., 2024), and spirometric evaluations of healthcare workers at three years confirmed persistent FEV_1_ and FVC reductions versus pre-pandemic values (Ippoliti et al., 2025).

In contrast to DLCO impairment, spirometric abnormalities are less frequently reported and tend to be milder. Across multiple prospective and retrospective studies, normal spirometry findings were documented in 72%–75.5% of survivors (Thomas et al., 2021; Chai et al., 2024), confirming that DLCO is the most sensitive PFT marker of post-COVID-19 pulmonary impairment. Most survivors exhibit mild-to-moderate DLCO reductions of 20–30% below predicted, consistent with residual alveolar-capillary membrane disruption and microvascular remodeling rather than complete fibrotic obliteration (Thomas et al., 2021).

Objectively measured functional capacity in post-COVID-19 survivors, assessed using the 6MWT, has also been significantly impaired. A retrospective study of 57 post-discharge patients found a mean 6MWT distance of 561.97 meters (range: 287–700 m), with shorter distances correlating with more severe acute illness and lower DLCO values (Watanabe et al., 2022). For reference, population normative values for healthy adults aged 40–60 years typically average 580–620 meters per ATS guidelines, indicating that the observed mean in this post-COVID cohort represents a clinically meaningful deficit below age-expected performance (Guidelines for the six-minute walk test, 2002). A three-year longitudinal cohort demonstrated only marginal improvement in 6MWT distance over time, while lung function abnormalities persisted in a substantial proportion of survivors (Han et al., 2024). Occupational and social reintegration are further affected: two-year follow-up data found that 57.5% of survivors required rehabilitation admission and 24.6% were unable to return to work (González et al., 2023), imposing a sustained socioeconomic cost on individuals, families, and healthcare systems.

## 6 Radiological findings on HRCT in post-COVID-19 survivors

High-resolution computed tomography (HRCT) has provided invaluable complementary evidence to PFT data, enabling direct visualization of structural pulmonary abnormalities. Up to 85% of patients undergoing HRCT following COVID-19 demonstrated pulmonary abnormalities, with bilateral involvement in 75% (Frija-Masson et al., 2020), underscoring the extent of parenchymal involvement even after apparent clinical recovery.

The radiological spectrum of post-COVID-19 abnormalities is diverse and evolves over time, with heterogeneous outcomes across studies. Ground-glass opacities (GGOs) and consolidation hallmarks of the acute inflammatory phase tend to partially resolve in many patients over months. An early 3-month assessment of 55 patients found that only 14 demonstrated persistent radiological impairment, reflecting the partial and variable resolution of CT findings in a subset of patients; this finding should be interpreted as illustrating the heterogeneity of early CT trajectories rather than as contradicting evidence of longer-term radiological burden (Vijayakumar et al., 2022). Larger and longer-term data consistently document a substantial proportion of survivors with residual abnormalities: a prospective observational study assessing 80 survivors over one year found radiological abnormalities in 56% at three months (Zhao et al., 2020), and a meta-analysis of one-year CT follow-up studies documented residual abnormalities in approximately 52–64% of patients across included cohorts (Hama Amin et al., 2022). A three-year longitudinal cohort further confirmed that HRCT abnormalities declined modestly from 46% to 36% over three years a trajectory of slow and incomplete resolution (Han et al., 2024).

Studies focusing specifically on mechanically ventilated ICU survivors have documented particularly high rates of persistent radiological changes. A prospective study of 54 mechanically ventilated patients admitted to ICU for COVID-19-related respiratory failure found significant and sustained HRCT abnormalities at six-month follow-up, with the severity and duration of mechanical ventilation independently predicting the degree of radiological change (Stoian et al., 2023).

An important distinction must be drawn within the spectrum of post-COVID-19 HRCT abnormalities. Not all persistent radiological changes represent established irreversible fibrosis; many findings particularly GGOs, ground-glass reticulation, and organizing pneumonia patterns may reflect ongoing but potentially reversible inflammatory activity or incomplete resolution of acute-phase changes. True irreversible pulmonary fibrosis, characterized by honeycombing, traction bronchiectasis, and architectural distortion, has been documented in approximately 12% of post-COVID-19 patients on HRCT (McDonald, 2021), while a broader meta-analysis found that 44.9% of post-COVID-19 patients developed any HRCT-visible pulmonary change (Alrajhi, 2023). Clinically, this distinction matters for prognosis and therapeutic decision-making: reversible changes may respond to anti-inflammatory rehabilitation or spontaneous resolution, while established fibrosis implies a requirement for long-term surveillance and potentially antifibrotic therapy.

Notably, even patients with mild infection who recovered without formal acute care may exhibit radiological and functional abnormalities at three months, including restrictive defects (14.4%) and small airway disease (8.4%), alongside persistent cough (60.4%) and dyspnea (55.6%) (Wei et al., 2020), underscoring that pulmonary sequelae are not confined to hospitalized individuals.

The relationship between HRCT findings and functional PFT outcomes is well established. Multi-parameter assessment studies including HRCT, PFTs, and the 6-minute walk test (6MWT) have demonstrated that DLCO is the most affected PFT parameter (52.6% impairment), that exercise capacity correlates inversely with HRCT severity, and that functional decline is greater in those with fibrotic versus non-fibrotic HRCT patterns (Watanabe et al., 2022). A three-year longitudinal cohort further demonstrated that HRCT abnormalities declined from 46% to 36% over three years, while DLCO abnormalities persisted in approximately 51% at the same timepoint (Han et al., 2024), confirming that functional and radiological recovery trajectories are not always concordant and that functional testing remains indispensable even when HRCT findings appear to improve.

## 7 Risk factors for persistent pulmonary dysfunction

A growing body of evidence has sought to characterize the demographic, clinical, and physiological determinants of long-term pulmonary impairment in COVID-19 survivors. **Table 2** summarizes the principal risk factors ranked by strength of available evidence.

**Table 2:** Principal risk factors for long-term pulmonary dysfunction in COVID-19 survivors, ranked by strength of evidence.

Rank	Risk factor	Proposed mechanism	Biologic plausibility	Consistency acute + chronic	Replication	Key references
1	ICU admission/mechanical ventilation	ARDS, DAD, fibroproliferative remodeling; prolonged immobility	High	Consistent	Multiple independent studies	González 2023; Stoian 2023; Berentschot 2024 (González et al., 2023; Stoian et al., 2023; Berentschot et al., 2024)
2	Older age (≥60 years)	Immunosenescence; reduced alveolar regenerative capacity; fibrotic susceptibility	High	Consistent	Multiple independent studies	Han 2024; Nurchis 2020; Stoian 2023 (Nurchis et al., 2020; Stoian et al., 2023; Han et al., 2024)
3	Pre-existing CLD (COPD, ILD, asthma)	Reduced functional reserve; amplified inflammatory burden; impaired mucociliary clearance	High	Consistent	Multiple independent studies	Zhang 2024; Calver 2023; Henry 2025 (Calver et al., 2023; Zhang et al., 2024; Henry et al., 2025)
4	Severity of acute pneumonia	Degree of parenchymal injury proportional to inflammation and alveolar-capillary disruption	High	Consistent	Moderate (fewer chronic-phase studies)	Salem 2021; Rosas 2025 (Salem et al., 2021; Rosas et al., 2025)
5	Obesity/Elevated BMI	Thoracic restriction; heightened systemic inflammation; impaired mucosal immunity	High	Consistent (acute); mixed (chronic)	Multiple independent studies	AlBahrani 2023; Alqahtani 2022; Lindgren 2022 (Alqahtani et al., 2022; AlBahrani et al., 2023)
6	Female sex	Immune response differences; hormonal modulation of alveolar inflammation; ACE2 hormonal regulation	Moderate	Mixed (conflicting across studies)	Multiple studies (conflicting)	Fernández-de-las-Peñas 2022; Kamal 2025; Moreno-Pérez 2021 (Fernández-de-las-Peñas et al., 2021; Moreno-Pérez et al., 2021; Kamal et al., 2025)
7	Diabetes mellitus	Impaired innate immunity; endothelial dysfunction; heightened inflammatory response; ACE2 dysregulation	High	Consistent (acute); Emerging (chronic)	Multiple studies	Alharbi 2021; Kamal 2025; Zhang 2024 (Alharbi et al., 2021; Zhang et al., 2024; Kamal et al., 2025)
8	Hypertension	ACE2 upregulation in RAAS-activated endothelium enhances viral entry; pre-existing endothelial dysfunction potentiates microvascular injury	High	Consistent (acute); Emerging (chronic)	Multiple studies	Alharbi 2021; Cai 2024; Althumiri 2021 (Alharbi et al., 2021; Althumiri et al., 2021; Cai et al., 2024)

CLD, chronic lung disease; COPD, chronic obstructive pulmonary disease; ILD, interstitial lung disease; DAD, diffuse alveolar damage; ICU, intensive care unit; ARDS, acute respiratory distress syndrome. Evidence strength is based on consistency across studies, sample size, and study design quality.

### 7.1 Acute disease severity and ICU admission

Acute disease severity and ICU admission represent among the strongest predictors of long-term pulmonary sequelae. Multiple cohort studies have demonstrated that patients requiring hospitalization—and particularly those admitted to the ICU or requiring mechanical ventilation—are at substantially elevated risk of sustained pulmonary dysfunction, including reduced DLCO, fibrotic HRCT changes, and persistent respiratory symptoms (González et al., 2023; Berentschot et al., 2024; Cai et al., 2024). Stoian et al. (2023) specifically documented that the degree and duration of mechanical ventilation in ICU-managed patients independently predicted the severity of HRCT changes at six-month follow-up (Stoian et al., 2023).

### 7.2 Older age

Advancing age is a consistent and biologically plausible risk factor. Older individuals exhibit immunosenescence, reduced regenerative capacity, and heightened susceptibility to fibroproliferative injury (Nurchis et al., 2020; Fernández-de-las-Peñas et al., 2022). and longitudinal data confirm that older age independently predicts persistent HRCT abnormalities including fibrotic changes (Alrajhi, 2023; Han et al., 2024).

### 7.3 Sex

Sex has emerged as a contested but frequently reported risk factor. Several studies have identified female sex as a predictor of long-term COVID-19 symptoms and complications (Moreno-Pérez et al., 2021; Sykes et al., 2021; Xiong et al., 2021), potentially reflecting differences in immune response profiles or hormonal modulation of inflammation. However, other cohort studies have not identified significant sex-based associations (AlBahrani et al., 2023; Calver et al., 2023), and the evidence remains conflicting, likely owing to differences in study design, follow-up duration, and comorbidity profiles.

### 7.4 Pre-existing chronic lung disease

Pre-existing CLD substantially amplifies the risk of post-COVID-19 pulmonary sequelae. COPD, asthma, interstitial lung disease, and pulmonary hypertension are each associated with more severe acute illness and higher rates of long-term complications (AlBahrani et al., 2023; Zhang et al., 2024). SARS-CoV-2 infection may itself precipitate *de novo* CLD, creating a cycle of progressive respiratory impairment in vulnerable individuals.

### 7.5 Obesity and body mass index

Obesity and elevated BMI have been implicated as risk factors for both severe acute COVID-19 and long-term pulmonary complications, principally through pulmonary restriction, heightened systemic inflammation, and impaired mucosal immunity (Fernández-de-las-Peñas et al., 2021; Alqahtani et al., 2022; Lindgren et al., 2022). Population-level data from Saudi Arabia confirmed significantly higher rates of severe COVID-19 in patients with BMI ≥40 kg/m² (Malik et al., 2022). However, the relationship between BMI and post-COVID pulmonary outcomes is less clearly established across all study populations, potentially owing to confounding by comorbidity burden and sample size limitations.

### 7.6 Hypertension

Hypertension has emerged as a clinically important risk factor for severe acute COVID-19 and, increasingly, for post-COVID-19 sequelae, with particular relevance to the Jizan region where hypertension was among the predominant comorbidities in ICU admissions (Alharbi et al., 2021). SARS-CoV-2’s primary binding target, ACE2, is upregulated in hypertensive patients with an activated renin-angiotensin-aldosterone system (RAAS), potentially amplifying viral entry into pulmonary endothelial cells. Hypertension-associated pre-existing endothelial dysfunction may further potentiate the SARS-CoV-2-driven microvascular injury cascade, creating a more severe and potentially more persistent V/Q mismatch. Epidemiological data from large-scale cohort analyses of post-acute sequelae consistently identify hypertension among the comorbidities associated with both higher acute severity and increased risk of long-term complications (Cai et al., 2024). A three-year PASC outcomes cohort (Cai et al., 2024) documented hypertensive patients as among those at elevated risk of persistent cardiopulmonary complications. These considerations make hypertension a priority surveillance target in follow-up programs for COVID-19 survivors, especially in populations with high hypertension prevalence such as Jizan.

## 8 Pulmonary rehabilitation: evidence and emerging strategies

The evidence base for pulmonary rehabilitation in post-COVID-19 survivors has grown considerably since the early pandemic period, with multiple studies demonstrating clinically meaningful benefits across functional, physiological, and psychological outcomes. **Table 3** summarizes the key published rehabilitation studies and their principal findings.

**Table 3:** Summary of key pulmonary rehabilitation studies in post-COVID-19 survivors.

Study	Design	n	Intervention	Key outcomes
Liu et al. (2020)	RCT	76	6-week structured respiratory rehabilitation vs. standard care	↑ DLCO; ↑ FVC; ↑ 6MWT; ↓ depression & anxiety scores
Szarvas et al. (2024) (Szarvas et al., 2024)	Observational quasi-experimental	100	2-week low-intensity program (breathing exercises + aerobic conditioning)	Sustained ↑ exercise performance & health status at 3-month follow-up
Haque & Sardar (2024)	Longitudinal cohort	104	Multidisciplinary program (aerobic + resistance + psychological support)	68% ↑ body function; 25% ↑ exercise capacity; 40% ↓ dyspnea

RCT, randomized controlled trial; DLCO, diffusing capacity of the lung for carbon monoxide; FVC, forced vital capacity; 6MWT, 6-minute walk test. ↑, improvement; ↓, reduction.

The mechanism by which structured pulmonary rehabilitation improves DLCO in COVID-19 survivors is likely multifactorial and remains an active area of investigation. Three principal pathways merit consideration. First, aerobic conditioning programs increase cardiac output, which enhances pulmonary capillary recruitment and blood flow to previously underperfused alveolar units, directly reducing V/Q mismatch and improving gas exchange (Szarvas et al., 2024). Second, respiratory muscle training and inspiratory threshold loading improve FVC and inspiratory capacity, increasing alveolar contact time with gas and potentially expanding the effective gas-exchange surface area. Third, emerging evidence suggests that sustained aerobic exercise may promote endothelial recovery and microvascular remodeling providing a speculative but biologically plausible pathway through which exercise could ameliorate the microthrombus-driven component of DLCO impairment (Kamal et al., 2025; Vanderbilt et al., 2025). These mechanisms are not mutually exclusive; it is most likely that the observed DLCO improvement in rehabilitated patients reflects a combination of improved perfusion, better respiratory mechanics, and partial endothelial recovery.

Evidence specifically supporting the effect of rehabilitation on 6MWT distance in COVID-19 survivors is now well established (Vranić et al., 2024). In a study of 80 post-COVID patients enrolled in a 3-week hospital-based pulmonary rehabilitation program, demonstrated statistically significant improvements in 6MWT parameters alongside improvements in FVC, FEV_1_, DLCO, and respiratory muscle strength (all p<0.05) (Szarvas et al., 2024). Spielmanns et al. (2022) prospectively enrolled 92 consecutive long-COVID patients in a 6-week individualized outpatient rehabilitation program and documented a clinically meaningful improvement in 6-minute walk distance as the primary endpoint, alongside improvements in dyspnea, fatigue, and quality of life (Spielmanns et al., 2022). These data, combined with the RCT evidence from Liu et al. (2020) and the longitudinal cohort evidence from Szarvas et al. (2024), demonstrate that pulmonary rehabilitation produces objective and clinically meaningful improvements across the full spectrum of post-COVID-19 impairment from DLCO and spirometric values to exercise capacity, 6MWT performance, and patient-reported outcomes.

However, challenges remain in translating these findings into clinical practice. The optimal duration, intensity, composition, and timing of rehabilitation programs have not been standardized. Published programs vary in duration from two to six weeks and in delivered components (breathing exercises, aerobic conditioning, resistance training, psychological support). Most trials have been conducted at single centers with small sample sizes and limited follow-up periods, constraining the generalizability of conclusions. Risk stratification frameworks incorporating PFT parameters, HRCT findings, symptom burden, and demographic variables could facilitate targeted triage of high-risk survivors toward early and intensive programs.

## 9 COVID-19 in Saudi Arabia and the Jizan region: a regional perspective

A dedicated regional perspective on Saudi Arabia is warranted for reasons directly germane to the pulmonary sequelae of COVID-19. As detailed in the Introduction, the Arabian Peninsula’s distinctive metabolic comorbidity profile including among the highest global prevalence rates of diabetes mellitus, obesity, and hypertension represents a constellation of risk factors that independently predict severe acute COVID-19 and impair long-term pulmonary recovery. Despite this, the current evidence base on post-COVID-19 pulmonary outcomes in Saudi Arabia is almost entirely symptom-focused and lacks objective PFT-based longitudinal data. This section synthesizes the available regional evidence and delineates the specific gaps that require investigation.

A national cross-sectional study of 314,821 post-COVID-19 survivors in Saudi Arabia found that 98.9% experienced at least one acute post-COVID symptom, with ongoing respiratory symptoms including shortness of breath (47%) remaining prevalent beyond the acute phase (AlRadini et al., 2022).

A broader observational study of 486 post-COVID-19 patients in Saudi Arabia, with up to two years of follow-up, confirmed persistence of breathing difficulties, fatigue, and anosmia; however, none of these national studies included objective pulmonary function measurements, preventing any characterization of the functional and radiological burden of long COVID in this population (Almalag et al., 2025). A population-based cross-sectional study of 2,063 survivors further identified physical condition abnormalities in 91.2%, depression in 77%, and post-COVID fatigue in 52% of the sample (Al-Johani et al., 2023). Female sex and the presence of comorbidities were consistently associated with adverse post-COVID outcomes across Saudi national studies.

Regional variation in disease severity has also been documented within Saudi Arabia. A retrospective cross-sectional analysis of Ministry of Health administrative data from 1,743 inpatients across 30 hospitals found ICU admission rates varying markedly across the country’s administrative regions, from 12% in the eastern province to approximately 88% in the northern region (Ribeiro Carvalho et al., 2024). The Jizan region in the southwestern province reported an ICU admission rate of 25% among confirmed COVID-19 cases the highest within its provincial cluster with the majority of ICU admissions occurring in patients with underlying diabetes and hypertension (Ribeiro Carvalho et al., 2024).

Given the well-established relationship between ICU admission and long-term pulmonary impairment, Jizan’s elevated acute disease severity predicts a corresponding population of survivors at heightened risk of DLCO reduction, restrictive ventilatory defects, and fibrotic HRCT changes. The near-complete absence of formal PFT-based post-COVID-19 assessments from this region constitutes a significant gap in the regional clinical literature that should be addressed through prospective, multicenter surveillance programs.

## 10 Discussion

This narrative review synthesizes the current state of evidence regarding the long-term pulmonary consequences of SARS-CoV-2 infection, drawing on an extensive and growing body of international literature spanning epidemiological, pathophysiological, functional, radiological, and clinical dimensions.

### 10.1 Impaired gas exchange as the hallmark deficit

Across the breadth of PFT-based studies reviewed, reduction in DLCO below 80% of predicted value emerges as the most consistently identified and clinically significant long-term pulmonary abnormality in COVID-19 survivors. Unlike spirometric parameters which are frequently preserved or only mildly reduced DLCO reflects the functional integrity of the alveolar-capillary unit and is uniquely sensitive to the microvascular injury and interstitial remodeling that characterize COVID-19 sequelae. DLCO impairment has been reported across diverse populations, timepoints, and disease severity strata, with prevalence estimates centering between 40% and 50% at three to six months, and only modest longitudinal improvement over two to three years of follow-up (Cornelissen et al., 2024; Han et al., 2024; Iversen et al., 2024). This pattern is consistent with residual alveolar-capillary membrane damage rather than acute inflammatory injury alone, suggesting a structural substrate that may not fully resolve in a meaningful proportion of survivors (do Espírito Santo et al., 2020; Nicolai et al., 2020; Kamal et al., 2025; Vanderbilt et al., 2025; Whitcomb et al., 2026).

### 10.2 Radiological abnormalities, fibrosis, and the functional interface

Restrictive ventilatory patterns predominate in post-COVID-19 PFT assessments, a finding mechanistically coherent with the fibroproliferative response to SARS-CoV-2-mediated alveolar injury. However, an important distinction must be maintained between persistent radiological abnormalities and established irreversible pulmonary fibrosis. The majority of post-COVID-19 HRCT changes including GGOs, organizing pneumonia patterns, and non-fibrotic reticulation may represent potentially reversible or stabilized post-inflammatory changes rather than established fibrosis. True irreversible fibrosis, characterized by honeycombing and architectural distortion, is documented in approximately 12% of survivors in well-characterized cohorts (McDonald, 2021), whereas the broader category of any persistent HRCT abnormality encompasses up to 44.9% (Alrajhi, 2023). Clinically, the functional consequence reduced DLCO and restricted lung volumes may be similar in the short term regardless of reversibility, but the prognostic and therapeutic implications differ substantially. This distinction should inform both the interpretation of follow-up HRCT findings and the selection of therapeutic interventions.

### 10.3 Geographic and demographic underrepresentation

The current evidence base is dominated by studies from Europe, East Asia, and North America. Populations in the Middle East, Sub-Saharan Africa, and South Asia are largely underrepresented., This geographic gap is particularly notable given that regional variation in factors such as ambient air quality, smoking patterns, the prevalence of diabetes and obesity, and potential ethnic differences in ACE2 expression may all modulate pulmonary vulnerability and recovery. Data from Saudi Arabia provide some regional context but remain primarily descriptive and symptom-focused, with a paucity of objective PFT-based longitudinal assessments conducted under standardized conditions. Targeted regional research is urgently needed.

### 10.4 Conflicting evidence on risk factors and therapeutics

The relationship between sex and long-term COVID-19 outcomes remains conflicting in the international literature. While several studies identify female sex as an independent risk factor for long COVID (Moreno-Pérez et al., 2021; Petersen et al., 2021; Calver et al., 2023), others have found no significant sex-based association (Petersen et al., 2021; Calver et al., 2023). A recent four-year prospective cohort study conducted in Saudi Arabia (n=816) confirmed female sex as an independent predictor of long COVID (aOR 1.8, p=0.01) and diabetes mellitus as the second strongest predictor (aOR 2.1, p=0.005), while obesity was not independently significant after multivariable adjustment providing important regional evidence that reconciles conflicting findings (Vranić et al., 2024). Similarly, the relationship between obesity and chronic-phase DLCO impairment is less consistently established than its role in acute disease severity (Althumiri et al., 2021; Fernández-de-las-Peñas et al., 2021; Alqahtani et al., 2022). The evidence on corticosteroid use in the post-acute phase is uniformly negative across studies conducted through 2024: while corticosteroids demonstrate clear benefit during acute inflammation, studies in the post-inflammatory recovery phase have not shown consistent improvements in DLCO, supporting the conclusion that mechanisms driving chronic pulmonary impairment differ substantially from those operative in acute disease (Salem et al., 2021; Thomas et al., 2021).

### 10.5 Limitations of the current literature

Despite the volume of published studies, several methodological limitations constrain conclusions. Most critically, the near-universal absence of pre-infection baseline PFT data precludes definitive attribution of observed impairments to COVID-19 itself, as opposed to pre-existing or coincidental pulmonary pathology. Marked heterogeneity in study designs including variable follow-up intervals, differing inclusion criteria, and inconsistent definitions of key outcomes limits cross-study comparability. Selection bias is a recurrent concern, particularly in studies restricted to hospitalized patients or those motivated to seek follow-up care, potentially overestimating the prevalence of impairment in the general survivor population. Furthermore, the absence of matched non-COVID control groups in most studies renders it difficult to disentangle COVID-19-specific effects from the effects of aging, comorbidity progression, and deconditioning during convalescence.

### 10.6 Future research directions

Priority areas for future investigation include the following. Prospective longitudinal studies with pre-infection PFT baselines and matched non-COVID control groups are needed to precisely define the physiological consequences of SARS-CoV-2 infection on pulmonary function independently of pre-existing pathology and to characterize the specific contribution of microvascular thrombosis versus fibroproliferative remodeling to DLCO impairment. Multicenter designs incorporating diverse geographic and demographic populations particularly from underrepresented regions such as the Arabian Peninsula are required to generate evidence applicable to the global survivor population and relevant to clinical guidelines across health systems with different metabolic comorbidity burdens. Standardized and harmonized PFT protocols and HRCT grading systems are essential to substantially improve cross-study comparability and the reliability of meta-analytic conclusions regarding the prevalence and trajectory of specific abnormalities. Precision-based risk stratification tools incorporating PFT parameters, imaging findings, symptom burden, and demographic variables including hypertension, diabetes, age, and sex should be developed and validated to guide individualized intensity and composition of post-COVID follow-up programs. Finally, rigorously designed and adequately powered pulmonary rehabilitation trials with objective endpoints including 6MWT, DLCO, and serial HRCT are needed to definitively establish the most effective rehabilitation modalities, optimal timing, and duration of interventions across defined risk subgroups and geographic populations.

## 11 Conclusion

The long-term pulmonary sequelae of SARS-CoV-2 infection constitute a clinically significant and incompletely understood dimension of the COVID-19 pandemic’s enduring burden. The available evidence robustly establishes that a substantial proportion of survivors develop persistent pulmonary dysfunction characterized principally by reduced DLCO, restrictive ventilatory defects, and fibrotic radiological changes. These abnormalities persist in many individuals for two years or longer, with only modest spontaneous recovery. ICU admission, older age, pre-existing CLD, and obesity have been identified as the strongest risk factors. Pulmonary rehabilitation has demonstrated clinically meaningful benefit. Significant gaps persist in the literature particularly regarding populations in the Arabian Peninsula and broader developing world necessitating targeted, methodologically rigorous regional investigations. Structured, longitudinal, region-specific investigations incorporating objective PFT assessments are essential to inform evidence-based clinical guidelines and mitigate the ongoing public health burden of post-COVID-19 pulmonary disease.
